# Origins and conservation of topological polarization defects in resonant photonic-crystal diffraction

**DOI:** 10.1515/nanoph-2024-0514

**Published:** 2025-01-03

**Authors:** Xuefan Yin, Takuya Inoue, Chao Peng, Susumu Noda

**Affiliations:** Department of Electronic Science and Engineering, Kyoto University, Kyoto-Daigaku-Katsura, Nishikyo-ku, Kyoto, 615-8510, Japan; State Key Laboratory of Advanced Optical Communication Systems and Networks, School of Electronics, & Frontiers Science Center for Nano-optoelectronics, 12465 Peking University, Beijing, 100871, China; Peng Cheng Laboratory, Shenzhen, 518055, China

**Keywords:** photonic crystal, topological charge, polarization defect

## Abstract

We present a continuative definition of topological charge to depict the polarization defects on any resonant diffraction orders in photonic crystal slab regardless they are radiative or evanescent. By using such a generalized definition, we investigate the origins and conservation of polarization defects across the whole Brillouin zone. We found that the mode crossings due to Brillouin zone folding contribute to the emergence of polarization defects in the entire Brillouin zone. These polarization defects eventually originate from the spontaneous symmetry breaking of line degeneracies fixed at Brillouin zone center or edges, or inter-band coupling caused by accidental Bloch band crossings. Unlike Bloch states, the polarization defects live and evolve in an unbound momentum space, obeying a local conservation law as a direct consequence of Stokes’ theorem, but the total charge number is countless.

## 1 Introduction

Polarization defects [1], [2], [3], [4], [5], [6], [7], [8], [9], [10], [11], [12], [13] are exotic phenomena that can happen in both real and momentum space at which one or two components that compose the light’s polarization are ill-defined, corresponding to some special points on the Poincaré sphere, such as the north or south poles which represent the circular-polarized states (CPs) [9], [14], [15], [16] and the amplitudes singularity at the sphere center that is related to bound states in the continuum (BICs) [17], [18], [19], [20], [21], [22], [23], [24]. Polarization defects bridge the underlying non-trivial physics in singular optics [25], [26], [27] and non-Hermitian systems [28], [29], [30], [31], [32] to the characteristics of the far-field radiation, thus enabling many applications such as high-*Q* cavities [33], vortex beam generators [34], and chiral devices with circular dichroism [35], [36]. In particular, from the view of topological photonics [37], [38], [39], [40], [41], [42], [43], the polarization defects in photonic crystal (PC) slabs can be characterized by quantized topological charges [44], [45], [46], [47], [48], [49]. For example, the BICs carry integer topological charges; the CPs and some paired exceptional points (EPs) [50], [51], [52], [53], [54], [55], [56] possess half-integer charges. The topological charge provides a vivid picture to depict and manipulate the far-field radiation and paves the way to rich consequences such as merging BICs [57], [58], [59] and unidirectional guided resonances (UGRs) [60], [61], [62].

Although topological charges establish a valid interpretation of polarization defects, such a picture is still incompetent in clarifying several important elusiveness, mainly because it is defined on the far-field radiation, and thus, their evolution is limited inside the light cone. For instance, it is not clear how the polarization defects originate in physics and how they evolve in the whole Brillouin zone (BZ). Besides, as the footstone of the topological charge’s theory, the conservation of topological charges in momentum space has been widely recognized. Nevertheless, it remains elusive what the physical origin of this conservation law is, and whether or not any global conservation of topological charges exists if taking all types of polarization defects into account.

To address the questions mentioned above, we first generalize the definition of topological charge to near-field context in this letter, which is not only consistent with the conventional definition in characterizing the radiative waves but can also depict the topological features in evanescent waves. As a result, such a generalized topological charge is valid in the entire BZ and can be applied to any diffraction orders including the non-radiative ones. Then, we reveal that in periodic structure, owing to BZ folding, polarization defects are eventually resulted from the line degeneracy fixed at BZ center or edges, or inter-band coupling near the accidental Bloch band crossings. Moreover, we point out that the polarization defects in a given diffraction order live in an unbound momentum space rather than the reduced BZ. Therefore, we need to consider the entire BZ to track their dynamic evolution, which is quite different from the Bloch states. Nevertheless, the topological charges always obey the local conservation law due to Stokes’ theorem, but the total charge number is countless.

## 2 Results and discussions

### 2.1 Generalizing the definition of topological charges to full field

**Figure 1:**
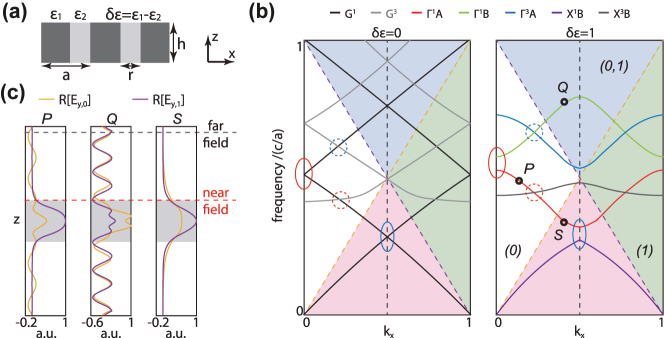
Resonant diffraction orders in periodic PC slab. (a) Schematic of the 1D PC slab. (b) Band diagrams of the effective homogeneous slab (*δɛ* = 0, left panel) and the periodic PC slab (*δɛ* = 1, right panel), with *r*/*a* = 0.4 and *h*/*a* = 1.2. Solid circles: crossings between the waveguide modes of the same order; dashed circles: accidental crossings between different waveguide modes. Indices in each region indicate the radiative diffraction orders. Dashed orange/purple lines: light lines for the 0th/1st diffraction orders. Dashed grey line: BZ edge. (c) Profiles of 0th (orange) and 1st (purple) diffraction orders for P , Q and S modes shown in (b). Grey shading represents the slab.

To elaborate on our findings, we start from a 1D dielectric slab as shown in Figure 1a and consider the transverse-electric (TE) modes with main components of (*H*_*x*_, *E*_*y*_, *H*_*z*_) for simplicity, while the discussion upon the 2D PC can be found in Supplementary materials. We note that the other components such as *E*_*x*_ are not zero, especially when it deviates from the *k*_*x*_ axis. When the alternating dielectric layers in the PC have the same relative permittivities (*ɛ*_1_ = *ɛ*_2_), the slab degrades to a conventional homogeneous slab waveguide, supporting several waveguide modes such as *G* ^1^ (black line) and *G* ^3^ (grey line) shown in left panel of Figure 1b. By assuming an artificial periodic modulation of permittivity *δ*_*ɛ*_ = *ɛ*_1_ − *ɛ*_2_ along *x* direction with a crystal constant of *a*, an artificial Bloch wave vector *k*_*x*_ *β*_0_ can be defined, where *β*_0_ = 2*π*/*a* is the reciprocal lattice constant. As a result, the energy dispersion curves of waveguide modes fold back to the reduced BZ and thus result in varieties of band crossings. Obviously, there are two types of crossings due to the folding. First, the waveguide modes of the same order (i.e. *G* ^1^ propagating towards the left and the right) fold to cross at the BZ center or edges (solid circles, left panel); second, waveguide modes of different orders (i.e. *G* ^1^ and *G* ^3^) may cross with each other accidentally (dashed circles, left panel). The crossings in the first case are inevitable as long as the periodic modulation is applied. As a comparison, the crossings in the second case are accidental and tunable if the frequencies of waveguide modes vary, and thus can be avoided totally by choosing proper parameters. Note that when *δ*_*ɛ*_ = 0, the folded waveguide modes simply cross with each other without coupling due to the orthogonality.

Then, we introduce nonzero *δ*_*ɛ*_ to turn the homogeneous slab into a PC slab. Due to the periodic modulation of permittivity, if symmetries allow, two crossed waveguides modes could couple with each other. Specifically, for first crossing scenario mentioned above, waveguide modes of the same order couple with themselves, get anti-crossed to split the photonic bandgap, forming series of Bloch energy bands (solid circles, right panel of Figure 1b). For the second case, inter-band coupling may emerge between different Bloch bands (dashed circles, right panel of Figure 1b), giving rise to different crossing types (crossing or anti-crossing) based on the coupling strength [62]. The crossings and coupling between energy bands not only result in fancy band structures, but also provide possibilities to manipulate the far-field radiation, and thus create abundant polarization defects by hybridizing multi radiation channels belonging to different Bloch modes.

To investigate how to generate polarization defects through the band crossings, we first propose a general picture to depict the radiation and polarization of Bloch modes in PC slab. According to the Bloch’s theorem, Bloch modes in periodic structure can be decomposed as a series of diffraction orders [63], [64]:

(1) $$\begin{array}{ll} E_{y}\left(r\right) & =\underset{m}{\sum }E_{y,m}\left(z\right){\text{e}}^{\text{i}\left(m-k_{x}\right)\beta_{0}x-ik_{y}\beta_{0}y}\\ & =\underset{m}{\sum }E_{y,m}\left(z\right){\text{e}}^{\text{i}g_{m}\cdot r} \end{array}$$

with *k*_*y*_ *β*_0_ is the continuous non-Bloch wave vector along *y* direction. Accordingly, the in-plane momentum of *m*th diffraction order is given by **g**_**m**_ with $k_{\|}=\left(k_{x}{\hat{e}}_{x}+k_{y}{\hat{e}}_{y}\right)\beta_{0}$ as the offset wavevector to the Γ point. The diffraction orders can be either radiative or evanescent, determined by whether its in-plane momentum falls into the light cone as |**g**_**m**_| < *ω*/*c* or not. This criteria gives light lines for each diffraction orders, such as dashed orange and purple lines for the 0th and 1st order in Figure 1b. These light lines divide the *ω* − *k*_*x*_ diagram into several regions. We color these regions in Figure 1b to distinguish whether diffraction orders (*m*_1_, *m*_2_, …) are radiative or not. We pick three Bloch modes *P*, *Q*, *S* in different regions as examples, whose profiles are shown in Figure 1c. Obviously, the number of radiative diffraction orders differs in different regions. In any regions with only one radiative channel, such as white region above the 0th lightline (left panel, Figure 1c), only one diffraction order (i.e. 0th order) is radiative, referred as the conventional far-field radiation channel. Elimination of such a radiation channel gives rise to the emergence of BICs, and the characteristics of polarization defects in such a channel can be interpreted by utilizing the topological charge picture. As for the regions with multi radiation channels, such as blue region where both 0th and 1st diffraction orders are radiative (middle panel, Figure 1c), topological charges can be applied independently to depict the radiation geometry of each individual channel [65]. In pink regions below all the light lines (right panel, Figure 1c), all the diffraction orders become evanescent and vanish in the far-field, making the conventional definition of radiation as well as topological charges invalid.

**Figure 2:**
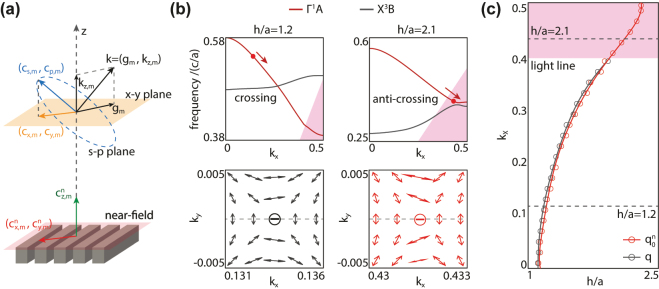
Near-field topological charge. (a) Schematic of far-field and near-field polarization. Black arrow: 3D radiative wave vector **k** = (**g**_*m*_, *k*_*z*,*m*_); blue dashed plane: far-field s−p plane perpendicular to **k**; blue arrow: far-field radiation in s−p plane; yellow plane: far-field x−y plane; yellow arrow: projected far-field radiation in x−y plane; red plane: near-field plane located at upper surface of the PC slab; red arrow: near-field components in x−y plane; green arrow: *z* component of near-field wave. (b) Top panels: band structures of $\Gamma_{A}^{1}$ (red line) and $X_{B}^{3}$ (gray line) modes with slab thickness of *h*/*a* = 1.2 and 2.1, respectively. A polarization defect (red dot) is found in $\Gamma_{A}^{1}$ band. When increasing the slab thickness, the polarization defect evolves along *k*_*x*_ axis (red arrow). Bottom panels: conventional topological charge *q* and generalized topological charge $q_{0}^{n}$ carried by the polarization defect with slab thickness of *h*/*a* = 1.2 and 2.1, respectively. (c) Evolution of conventional (grey dotted line) and generalized (red dotted line) topological charge carried by the polarization defect.

Accordingly, the topological charges picture is only valid where radiative channels can be well-defined. However, the band crossings can happen everywhere in the whole BZ. To figure out how band crossings contribute to the emergence of polarization defects, we generalize the definition of topological charges upon non-radiative diffraction orders. Specifically, as shown in Figure 2a, if *m*th diffraction order is radiative, the far-field polarization (*c*_*s*,*m*_, *c*_*p*,*m*_) is generally elliptical and transverse to the 3D wave vector **k** = (**g**_*m*_, *k*_*z*,*m*_), where $k_{z,m}=\sqrt{\omega_{b}^{2}/c^{2}-|g_{m}{|}^{2}}$ is the vertical wave vector of this diffraction order and *ω*_*b*_ is the frequency of Bloch mode. Due to the transverse-wave nature of the radiation, polarization can be projected from s−p plane (dashed blue plane) onto the x−y plane (yellow plane) in an appropriate way with the polarization information preserved [24], [65], giving a reduced far-field radiation (*c*_*x*,*m*_, *c*_*y*,*m*_) in x−y plane. We note that, the radiation is actually dependent upon the 3D wave vector **k**. However, since one diffraction order is just a constituent of the Bloch mode, wave vector **k** is fully determined by the in-plane wave vector **k**_‖_. Therefore, we consider the characteristics of diffraction orders with respect to **k**_‖_ not the **k**.

Then, we consider the near-field waves $\left(c_{x,m}^{n},c_{y,m}^{n},c_{z,m}^{n}\right)$ , defined as the complex amplitude of diffraction orders at near-field position (red plane, Figure 2a). For radiative diffraction, since it’s continuous and uniform at any *z* position outside the slab, near-field waves $\left(c_{x,m}^{n},c_{y,m}^{n}\right)$ is exactly equivalent to the far-field one (*c*_*x*,*m*_, *c*_*y*,*m*_), and thus can be employed directly to depict the polarization. For non-radiative diffraction, vertical wave vector *k*_*z*,*m*_ becomes an imaginary number, and the far-field radiation vanishes. However, the transverse condition still holds for near-field waves (see Supplementary materials for details), ensuring that the information of component $c_{z,m}^{n}$ is totally encoded in other two components $c_{x,m}^{n}$ and $c_{y,m}^{n}$ . Therefore, we can still employ the near-field waves $\left(c_{x,m}^{n},c_{y,m}^{n}\right)$ to present the polarization, consistent with the radiative case. In other words, the near-field polarization defined by $\left(c_{x,m}^{n},c_{y,m}^{n}\right)$ is valid for any diffraction order *m* in the whole BZ, regardless they are radiative or evanescent. Accordingly, we can define the general topological charges in near-field waves as:

(2) $$q_{m}^{n}=\frac{1}{2\pi }\underset{C}{\oint }dk_{\|}\cdot \nabla_{k_{\|}}\theta_{m}^{n}\left(k_{\|}\right)$$

Here, $\theta_{m}^{n}\left(k_{\|}\right)$ is the orientation angle of near-field polarization $\left(c_{x,m}^{n},c_{y,m}^{n}\right)$ of *m*-order diffraction. Obviously, the conventional topological charge *q* defined in the far-field polarization is a subset of $q_{m}^{n}$ . In the following discussions we refer to them both as the “topological charges”.

The above continuative definition of $q_{m}^{n}$ allows us to learn how polarization defects evolve in the whole BZ. To show this fact, we take the $\Gamma_{A}^{1}$ band in Figure 2b as an example. This band spreads along the Γ-*k*_*x*_ direction and get below the 0th light line around the *X* point. As shown in left panel of Figure 2b, inside the light cone (white region), only 0th diffraction order is radiative, and a conventional integer topological charge (left bottom panel, Figure 2b) can be employed to describe a polarization defect (red dot) on it, corresponding to a tunable accidental BIC. When varying the parameter slab thickness *h*, this integer charge evolves robustly along the *k*_*x*_ axis (red arrow) until it enters into the pink region and thus drops out of the light cone. The evolution trajectory is shown in Figure 2c as the dotted grey line. Obviously, it is limited inside the light cone. As shown in the right panel of Figure 2b, when *h* increases to 2.1*a*, this polarization defect travels to *k*_*x*_ = 0.431 below the light line. In this case, it can’t be captured by the conventional topological charge any more since the conventional definition is only valid inside the light cone. As a comparison, we can employ the generalized topological charge defined in Eq. (2) to extract the evolution trajectory of this polarization defect outside the light cone. As shown in the right bottom panel of Figure 2b, the polarization defect carries a generalized charge of $q_{0}^{n}=-1$ in 0th diffraction order, consistent with the conventional one. The trajectory of this generalized charge is plotted in Figure 2c as the dotted red line. Obviously, inside the lightcone, the trajectories of conventional charge and generalized charge overlap well with each other, but the latter smoothly crosses the light line and evolves to the BZ edge (*k*_*x*_ = 0.5), verifying that generalized charge can be applied in the whole BZ. We note that, the slight deviation between the two trajectories inside the lightcone is resulted from the numerical error when we calculate the near-field polarization numerically through the Fourier transformation.

### 2.2 First crossing scenario: mode combination and formation of lattice charge

By utilizing the generalized topological charges, we investigate the physical origin of polarization defects, that is, how band crossings contribute to the emergence of polarization defects. We also discuss the conservation law that the generalized topological charges obey. As we stated above, in periodic PC slab there are two types of band crossings due to the BZ folding: inevitable crossings between the waveguide modes of the same order, and accidental Bloch bands crossings between waveguide modes of different orders. Both of them introduce the inter-mode couplings and can generate nontrivial polarization defects accordingly [23]. We first take waveguide mode *G* ^1^ as an example to investigate the first crossing scenario.

**Figure 3:**
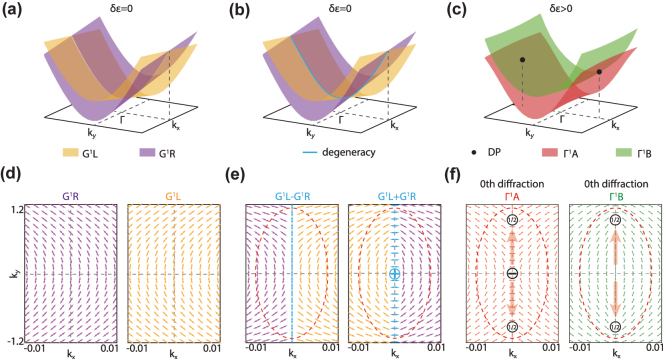
Generation of polarization defects from lattice charge. (a,b) Band structures when *δ*_*ɛ*_ = 0. Blue line: line degeneracy due to BZ folding. (c) Band structures when *δ*_*ɛ*_ > 0. (d,e) Polarization vector fields of waveguide modes $G_{R,L}^{1}$ (d) and their linear combinations along the line degeneracy (e) when *δ*_*ɛ*_ = 0. Nonzero lattice charge emerges due to mode combination at line degeneracy. (f) Polarization vector fields of Bloch modes $\Gamma_{A}^{1}$ and $X_{B}^{3}$ when *δ*_*ɛ*_ > 0.

Specifically, $G_{R}^{1}$ and $G_{L}^{1}$ denote the *G* ^1^ mode propagating towards the left and the right. Due to BZ folding, they cross with each other at Γ point [21], [23] (red circle, left panel of Figure 1b), forming a two-level system around the crossing point as:

(3) $${\hat{H}}_{0}=\omega_{0}+\eta k_{y}^{2}+\left(\Delta_{x}+\Delta_{y}k_{y}^{2}\right)\sigma_{x}+\xi k_{x}\sigma_{z}$$

where *ω*_0_ is the degenerate frequency of ${\text{G}}_{R,L}^{1}$ at Γ point, and *η*, *ξ* are coefficients associating with the dispersion. Parameters Δ_*x*,*y*_ describe the periodic permittivity modulation and thus depend on *δ*_*ɛ*_. For a homogeneous slab (*δ*_*ɛ*_ = 0), both Δ_*x*_ and Δ_*y*_ are zero, and thus $G_{R}^{1}$ and $G_{L}^{1}$ simply cross with each other at Γ point without coupling, whose energy bands are shown in Figure 3a. For waveguide modes, there are actually no diffraction orders. Nevertheless, we can calculate the near-field polarization vector fields of waveguide modes $G_{R,L}^{1}$ themselves according to Eq. (2), shown in Figure 3d (see Supplementary materials for details). Obviously, around the Γ point, there are no polarization defects for both two modes.

Although no polarization defect exists for individual $G_{R,L}^{1}$ , this crossing results in a line degeneracy at *k*_*x*_ = 0 along Γ-*k*_*y*_ direction (blue line, Figure 3b), where $G_{R,L}^{1}$ can hybridize with each other arbitrarily, giving rise to possible polarization singularities. Specifically, along the *k*_*y*_ axis (*k*_*x*_ = 0), two degenerate eigenvectors *ϕ*_1_ = [1,0]^*T*^ for $G_{L}^{1}$ and *ϕ*_2_ = [0,1]^*T*^ for $G_{R}^{1}$ can be solved according to Eq. (3). Due to the line degeneracy, any linear combinations of *ϕ*_1,2_ are still eigenvectors of Hamiltonian ${\hat{H}}_{0}$ along the *k*_*y*_ axis. Similar to the physics of spontaneous symmetry breaking [66], [67], to consistent with a real PC structure, the combinations should be determined according to the realistic periodic permittivity modulation. By assuming nonzero Δ_*x*,*y*_, the eigenvectors of ${\hat{H}}_{0}\left(k_{x}=0,k_{y}\right)$ are $\phi_{1,2}^{\prime }={\left[1,\pm 1\right]}^{T}$ . Accordingly, at the line degeneracy, the combinations of eigenvectors can be chosen as: $\phi_{1}\pm \phi_{2}={\left[1,\pm 1\right]}^{T}=\phi_{1,2}^{\prime }$ , corresponding to $G_{L}^{1}\pm G_{R}^{1}$ . By applying these combinations at the line degeneracy, we restructure the waveguide modes along the *k*_*y*_ axis, distinguish the two bands by their frequencies, and replot the near-field polarization vector fields in Figure 3e. Obviously, the generalized topological charge for $\left(G_{R}^{1}-G_{L}^{1}\right)$ is zero ( $q_{0,-}^{n}=0$ ) while an integer charge of $q_{0,+}^{n}=1$ can be found for $\left(G_{R}^{1}+G_{L}^{1}\right)$ . This nonzero charge doesn’t correspond to any physical polarization defect since it is directly resulted from a mathematical dealing: a linear combination of eigenmodes, and is a consequence of the spontaneous symmetry breaking of the line degeneracy. In other words, this nonzero topological charge is a footprint of the mode degeneracy in the polarization field and is determined only from the BZ folding brought by the periodic permittivity modulation. Thus, we denote it as the “lattice charge”.

By introducing the realistic periodic modulation to turn the homogeneous slab into a PC slab, the two waveguide modes couple with each other and the line degeneracy is lift to form two band-edge Bloch modes $\Gamma_{A,B}^{1}$ , whose band structures are shown in Figure 3c. For Bloch modes, varieties of diffraction orders can be defined according to Eq. (1). Here we take the 0th diffraction order as an example, whose near-field polarization vector fields for $\Gamma_{A,B}^{1}$ modes are shown in Figure 3f. Discussions about other diffraction orders can be found in Supplementary materials. Obviously, inside the region of interest (ROI, red dashed loop) around the Γ point, varieties of polarization defects carrying nontrivial topological charges are generated. Specifically, an integer charge of $q_{0}^{n}=-1$ can be found for $\Gamma_{A}^{1}$ mode, corresponding to a symmetry-protected BIC. Besides, a pair of Dirac points (DPs) can be found at $\left(k_{x}=0,k_{y}=\pm \sqrt{\Delta_{x}/\Delta_{y}}\right)$ for both $\Gamma_{AB}^{1}$ modes (black dots, Figure 3c), each carrying a half charge of $q_{0}^{n}=1/2$ (Figure 3f). As a result, we find that the total generalized topological charges inside the ROI for $\Gamma_{A,B}^{1}$ modes conserve to the lattice charge: $q_{0,-}^{n}=q_{0,A}^{n}=1/2+1/2-1=0$ and $q_{0,+}^{n}=q_{0,B}^{n}=1/2+1/2=1$ . This conservation originates from the fact that, the lattice change is intrinsically a consequence of eigenvector combination at line degeneracy, whose combination coefficients are chosen to be consistent with the case of realistic periodic modulation. Therefore, when homogeneous slab turns to real PC slab, the lattice charge turns into generalized topological charges carried by polarization defects in every diffraction orders (see Supplementary materials for details).

Similar line degeneracy also emerges at BZ edge (blue circle, left panel of Figure 1b). And nonzero lattice charge also emerges as the direct consequence of the mode combination and eventually turns into polarization defects for all the diffraction orders. We note that, as a mathematical concept, the lattice charge only depends on the assumed periodic modulation, or in other words, the lattice, and has no relationship with the concrete geometry of the unit cell of the PC slab. By changing the geometry or symmetry of the unit cell, the lattice charge may turns into different polarization defects, but the conservation law always holds. More discussions and examples can be found in Supplementary materials.

### 2.3 Second crossing scenario: inter-band coupling and local charge conservation

We take the $\Gamma_{A}^{1}$ mode and $X_{B}^{3}$ mode as examples to discuss how accidental Bloch band crossing gives rise to the emergence of polarization defects. As shown in Figure 1b, waveguide modes $G_{R}^{3}$ (grey line) and folded $G_{L}^{1}$ (black line) cross with each other due to BZ folding (dashed red circle). When introducing the periodic permittivity modulation, these two waveguide modes become Bloch bands $\Gamma_{A}^{1}$ and $X_{B}^{3}$ , between which the inter-band coupling occurs around the crossing point. As we stated before, this accidental crossing point is tunable when parameters vary. By increasing the slab thickness *h*, red shift of the frequency of $X_{B}^{3}$ mode makes the crossing point approach *X* point, until the $X_{B}^{3}$ band gets totally below the $\Gamma_{A}^{1}$ band and thus the crossing point disappears. On the other hand, decreasing the *h* results in blue shift of frequency of $X_{B}^{3}$ mode and thus the crossing point moves towards the Γ point. Considering the Q factor of $X_{B}^{3}$ is relatively low when *h* is small, we consider the coupling between $\Gamma_{A}^{1}$ and $X_{B}^{3}$ modes in 2D parameter space (*k*_*x*_, *k*_*y*_ = 0, *h*) near the *X* point.

**Figure 4:**
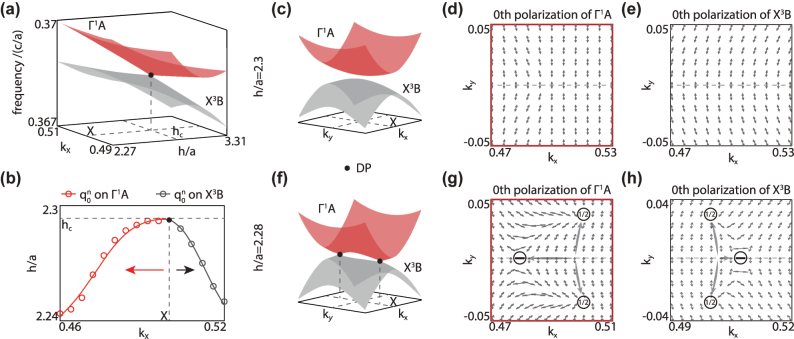
Generation of polarization defects from accidental inter-band coupling between different Bloch bands. (a) Coupling scenario of $\Gamma_{A}^{1}$ and $X_{B}^{3}$ modes in 2D parameter space (*k*_*x*_, *h*) around the *X* point. (b) Evolution of the polarization defects carrying integer topological charges on 0th diffraction order for $\Gamma_{A}^{1}$ (red dotted line) and $X_{B}^{3}$ (gray dotted line) modes. (c–h) Band structures, polarization vector fields for $\Gamma_{A}^{1}$ and $X_{B}^{3}$ when *h*/*a* = 2.3 (c–e) and *h*/*a* = 2.28 (f–h).

The band structures of $\Gamma_{A}^{1}$ and $X_{B}^{3}$ modes in such a parameter space are shown in Figure 4a. Obviously, around the *X* point, both the $\Gamma_{A}^{1}$ and $X_{B}^{3}$ modes are under the lightline and form a Hermitian two-level system. A DP (black dot) is found at (*k*_*x*_ = 0.5, *h*_*c*_ = 2.29*a*), giving a critical slab thickness *h*_*c*_ determines how bands get coupled. When *h* > *h*_*c*_ (i.e. *h*/*a* = 2.3, Figure 4c), two bands are gapped and there’s no inter-band coupling between them. Then, the polarization vector fields of 0th diffraction order are shown in Figure 4d and e for both two bands. Obviously, in the ROI (red box) around the *X* point, no polarization defects can be found. As a comparison, when *h* < *h*_*c*_ (i.e. *h*/*a* = 2.28, Figure 4f), two bands get crossed and coupled to each other, generating two DPs (black dots) at (*k*_*x*_ = 0.5, *k*_*y*_ = ±0.03). Due to the inter-band coupling, several polarization defects are spawned from the *X* point. As shown in Figure 4g and h, for 0th diffraction order, each DP generates a half-charge of $q_{0}^{n}=1/2$ . At the same time, an integer charge $q_{0}^{n}=-1$ appears for both two bands. It’s easy to find that inside the ROI, the total 0th topological charges are conserved for both bands, followed as $q_{0,all}^{n}=1/2+1/2-1=0$ which is the same as the uncoupled case.

In fact, for a specific diffraction order (i.e. 0th order here), inside the ROI around the *X* point, the total generalized topological charge carried by all the polarization defects generated from the second crossing scenario always conserves to zero. Different from the line degeneracy in the first crossing scenario, here the crossing between Bloch bands is accidental, local, and can be eliminated by simply adjusting the parameter of the PC slab to get two Bloch bands separated and uncoupled. When *h* > *h*_*c*_ (Figure 4c), the two bands are gapped inside and outside the ROI, and thus no polarization defects emerge. Then, when *h* get smaller than *h*_*c*_, two bands get crossed and coupled inside the ROI (Figure 4f), but still gapped outside the ROI. Since the polarization vector fields are smooth everywhere except the polarization defects, along the closed boundary of the ROI (i.e. red boxes in Figure 4d and g), the total winding number of polarization vector should be the same before and after the coupling, which equals to zero. Then, according to the Stokes’ theorem, it’s derived that the total topological charge of the polarization defects inside the ROI is always conserved to zero. By varying the parameters, the polarization defects may evolve away from the *X* point and leave the ROI. However, the local conservation still holds by enlarging the ROI to again include all these polarization defects generated from the second crossing scenario.

The evolution trajectories of two integer topological charges in Figure 4g and h are shown in Figure 4b, where the black dot denotes the DP which is the same with the one in Figure 4a. When slab thickness decreasing from critical value of *h*_*c*_, the two integer charges are spawned from the DP, and evolve towards the opposite direction: the integer charge on $X_{B}^{3}$ band moves away from the reduced BZ (black arrow), while the charge on $\Gamma_{A}^{1}$ band gradually approaches the Γ point (red arrow) and eventually falls into the light cone and become an accidental BIC, which is exactly the one shown in Figure 2. In other words, the accidental BIC in $\Gamma_{A}^{1}$ mode actually comes from the interband coupling happening at the BZ edge. In PC slab, the crossing behaviour between different Bloch bands is a common phenomenon and could result in varieties of polarization defects due to the inter-band coupling effect. Another example is $\Gamma_{B}^{1}$ and $\Gamma_{A}^{3}$ modes which cross and couple to each other (blue dashed circle, Figure 1b). Similar with the case we discuss above, by increasing the slab thickness *h*, the red shift of frequency of Bloch mode $\Gamma_{A}^{3}$ makes the crossing point move towards the Γ point, until the $\Gamma_{A}^{3}$ mode is totally below the $\Gamma_{B}^{1}$ mode and thus the crossing point disappears. The coupling between $\Gamma_{B}^{1}$ and $\Gamma_{A}^{3}$ modes around the Γ point gives rise to varieties of polarization defects including a Friedrich–Wintgen (FW) BIC [18]. And the local conservation due to the Stokes’ theorem also holds during the whole coupling process. The specific crossing behaviour is analyzed and presented in the Supplementary materials. Besides, TE band and transverse-magnetic (TM) band can also couple to each other in some cases to generate polarization defects [24].

### 2.4 Evolution of polarization defects in unfolded BZ

Combining the above discussions, we conclude that the band crossings in PC slab contribute to the emergence of polarization defects in any diffraction orders due to the BZ folding. These polarization defects carry nonzero generalized topological charges conserve to the lattice charge, or locally conserve to the trivial uncoupled case due to the Stokes’ theorem. Considering that the band crossings are universal and common in PC slab, it’s interesting and important to ask about whether the global conservation of all these topological charges in BZ exists or not. To answer this question, we note one important fact that, polarization defects belong to the feature of one specific diffraction order, but not the Bloch mode itself. According to the Bloch theorem, the reduced BZ is a compact and closed manifold for Bloch states, so that the discussions upon its characteristics can be limited inside the reduced BZ. As a comparison, a single diffraction order is not periodic over the whole momentum space. As for the polarization defects in a given diffraction order, the BZ is flattened and can’t be scrolled up. In fact, although originated from the BZ folding, the polarization defects can emerge everywhere in the entire BZ, not limited inside the reduced one. One example is the integer charge in $X_{B}^{3}$ band shown in Figure 4b which moves away from the reduced BZ When slab thickness *h* decreases.

According to Eq. (1), the *m*th diffraction order is exactly equivalent to *n*th order by translating a reciprocal vector of (*n* − *m*)*β*_0_ in momentum space due to the same in-plane wave vector:

(4) $$g_{m}\left(k_{x},k_{y}\right)=g_{n}\left(k_{x}+\left(n-m\right),k_{y}\right)$$

**Figure 5:**
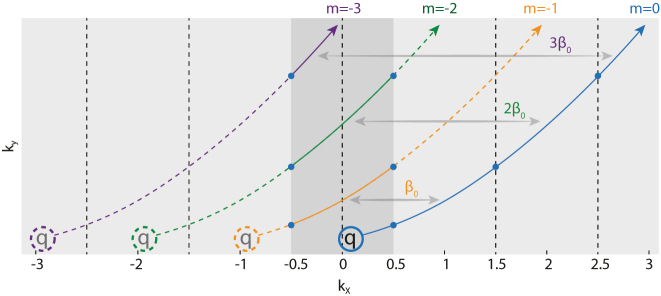
Polarization defects in unfolded BZ. Dark grey shading: reduced BZ; light grey shading: Entire BZ; solid arrows: schematic of evolution trajectories of polarization defects in different diffraction orders.

This equivalence indicates that, polarization defects in different diffraction orders can convert to each other. For example, as schematically shown in Figure 5, a polarization defect in 0th diffraction order (blue line) can convert into a −1th diffraction order (orange line) by translating a reciprocal vector of *β*_0_. Actually, it can also convert into any other −*m*th diffraction order by translating a reciprocal vector of *mβ*_0_. In other words, there are two ways to deal with the polarization defects in momentum space: taking all the polarization defects in multi diffraction orders into account but limited inside the reduced BZ, or focusing on polarization defects in only one diffraction orders (i.e. 0th order) but in the entire flattened BZ. The two pictures are equivalent to each other. Accordingly, there are also two viewpoints to deal with the global conservation of generalized topological charges.

In the first viewpoint, we consider the reduced BZ (dark grey shading, Figure 5), where exist infinite diffraction orders bearing possible polarization defects (solid lines, Figure 5). Obviously, the total number of polarization defects is countless since the number of diffraction orders is infinite. Moreover, it’s hard to deal with the topological charge right at the BZ edge (i.e. half charge in Figure 4g and h) if we only consider the reduced BZ. Also, it’s counter-intuitive to count for the total number of topological charges from different diffraction orders. As for the second viewpoint, we focus on only one diffraction order such as 0th order, distributed and evolving in entire BZ (blue line, Figure 5). And for polarization defects on any other diffraction order, we can convert them to defects in 0th order according to Eq. (4). In this way, the conservation question of topological charges turns out to ask whether the total 0th charges conserve or not in entire BZ. Obviously, the total number of polarization defects is still infinite, and thus it’s hard to derive a so-called “global” conservation in such a boundless momentum space. Nevertheless, the local conservation still holds to regulate the behaviour of topological charges. Specifically, as for the polarization defects rooted from the BZ folding, we have concluded that they are conserved with the lattice charge as a consequence of mode combination in line degeneracy. As for the polarization defects raised by accidental Bloch band crossings, they are actually local wrinkles on polarization vector field. Due to the Stokes’ theorem, they are always conserved to uncoupled case, which is zero.

## 3 Conclusions

To summarize, we generalize the definition of topological charge to any resonant diffraction orders regardless they are radiative or evanescent, which allows us to track and discuss the origins and conservation of polarization defects across the entire BZ. We found that in periodic PC structure, band crossings due to the BZ folding result in the emergence of polarization defects, which originate from the spontaneous symmetry breaking of the line degeneracy in BZ edge or center, or inter-band coupling between accidentally crossed Bloch bands. The dynamic behaviour of these polarization defects can be depicted by generalized topological charges, which locally conserve to lattice charges or trivial uncoupled case, respectively. Different from Bloch state, polarization defects evolve in unbound momentum space. We conclude that topological charge locally conserves owing to Stokes’ theorem, but the total number of charges in entire BZ is countless. Our work proposes a universal picture of polarization defects in any diffraction orders not just the radiative one. With this novel picture, it’s possible to realize topological charges in near-field and non-radiative channels, promote the conventional topological radiation theory from far field to the full field, and tailor the near-field diffraction arbitrarily from a topological perspective. Our theory is potentially powerful in boosting exotic phenomena about versatile light manipulation, such as near-field light confinement and enhancement, and near-field beam morphing and steering, thus benefiting various optoelectronic applications such as near-field illumination, near-field vortex beam generator, one-way wave guide and on-chip circulator.

## Supplementary Material

Supplementary Material Details
