# Supplementary material for: Origins and conservation of topological polarization defects in resonant photonic-crystal diffraction
Source: Nanophotonics. 2025 Jan 3;14(1):95–105. doi: 10.1515/nanoph-2024-0514 (PMC11744457; doi:10.1515/nanoph-2024-0514)
Supplement: Supplementary file 1 — Supplementary Material Details [file j_nanoph-2024-0514_suppl_001.pdf]

Supplementary Information – Origins and conservation of topological polarization defects in resonant photonic-crystal diffraction

Xuefan Yin[1], Takuya Inoue[1], Chao Peng[2*], Susumu Noda[1*]

[1] *Department of Electronic Science and Engineering, Kyoto University, Kyoto-Daigaku-Katsura, Nishikyo-ku, Kyoto 615-8510, Japan*

[2] *State Key Laboratory of Advanced Optical Communication Systems and Networks, School of Electronics, & Frontiers Science Center for Nano-optoelectronics, Peking University, Beijing, 100871, China*

1 The transverse condition of the near-field radiation

In this section, we present a proof of the transverse condition of the near-field radiation. We start from the general coupled-wave theory (CWT) framework[1]. Under this framework, the eigenstate $\mathbf{E}(\mathbf{r})$ and permittivity $\varepsilon(\mathbf{r})$ of the photonic crystal (PC) slab can be expanded in a plane-wave basis as:

$$E_{x,y} = \sum_{m,n} E_{x,y;m,n} e^{im\beta_0 x + in\beta_0 y} e^{-i\mathbf{k}_{/\!/}\mathbf{r}} \qquad \varepsilon(\mathbf{r}) = \xi_0(z) + \sum_{\substack{m\neq 0\\ n\neq 0}} \xi_{m,n}(z) e^{im\beta_0 x + in\beta_0}. \tag{S1}$$

Here, $\mathbf{k}_{/\!/} = k_x\beta_0\hat{x} + k_y\beta_0\hat{y}$ with $k_{x,y}$ denoting the Bloch's wavevector; $\beta_0 = 2\pi/a$ is the modulus of the primitive wave vectors in momentum space, and a is the lattice constant; m and n are integer numbers representing the order of Fourier expansion coefficient; ξ_0 is the averaging permittivity of the slab, given by $\xi_0 = f\varepsilon_1 + (1-f)\varepsilon_2$, where $f = r/a$ is the filling factor. These plane waves are exactly the resonant diffraction orders we described in the main-text. The coupling between individual diffraction orders can be described as:

$$\left[\frac{\partial^2}{\partial z^2} + k_0^2\xi_0(z) - (m_x^2 + n_y^2)\beta_0^2\right](n_y E_{x;mn} - m_x E_{y;mn}) = -k_0^2 \sum_{\substack{m'\neq m\\ n'\neq n}} \xi_{\substack{m'\neq m\\ n'\neq n}} (n_y E_{x;m'n'} - m_x E_{y;m'n'}) \tag{S2}$$

$$\left[\frac{\partial^2}{\partial z^2} + k_0^2\xi_0(z)\right](m_x E_{x;mn} + n_y E_{y;mn}) + \xi_0(z)\frac{\partial}{\partial z}E_{z;m'n'} = -k_0^2 \sum_{\substack{m'\neq m\\ n'\neq n}} \xi_{\substack{m'\neq m\\ n'\neq n}} (m_x E_{x;m'n'} + n_y E_{y;m'n'}) \tag{S3}$$

$$\left[k_0^2\xi_0(z) - (m_x^2 + n_y^2)\beta_0^2\right] E_{z;mn} + i\beta_0\frac{\partial}{\partial z}(m_x E_{x;mn} + n_y E_{y;mn}) = -k_0^2 \sum_{\substack{m'\neq m\\ n'\neq n}} \xi_{\substack{m'\neq m\\ n'\neq n}} E_{z;m'n'} \tag{S4}$$

where $m_x = m - k_x$ and $n_y = n - k_y$. According to equations above, we conclude the transverse condition as:

$$\xi_0(z)\left[(im_x\beta_0)E_{x;mn} + (in_y\beta_0)E_{y;mn}\right] + \xi_0(z)\frac{\partial}{\partial z}E_{z;mn}$$
$$= -\sum_{\substack{m'\neq m\\ n'\neq n}} \xi_{\substack{m'\neq m\\ n'\neq n}} \left\{(im_x\beta_0)E_{x;m'n'} + (in_y\beta_0)E_{y;m'n'} + \frac{\partial}{\partial z}E_{z;m'n'}\right\} \quad \text{(S5)}$$

Note that Eq. S5 exactly corresponds the transverse condition $\nabla \cdot [\xi(\mathbf{r})\mathbf{E}(\mathbf{r})] = 0$. Obviously, Eq. S5 holds at arbitrary position z. Outside the PC slab, the permittivity modulation ξ_{mn} becomes zero, and thus the transverse condition for each diffraction order becomes:

$$i\beta_0\left[m_x E_{x;mn}(z) + n_y E_{y;mn}(z)\right] + \frac{\partial}{\partial z}E_{z;mn}(z) = 0 \qquad \text{for } |z| > z_{PC} \quad \text{(S6)}$$

Eq. S6 indicates that for any diffraction order outside the slab, only two components are independent. For radiative diffraction order, the two independent components are the $(E_{s;m}, E_{p;m})$ in $s - p$ plane perpendicular to the radiative vector $\mathbf{k} = \left(m_x\beta_0, n_y\beta_0, k_z = \sqrt{k_0^2\xi_0^z - (m_x^2 + n_y^2)\beta_0^2}\right)$. As shown in Fig. 2 in the main-text, we can use their complex amplitudes $(c_{s;m}, c_{p;m})$ to depict the far-field polarization. However, for non-radiative diffraction order, k_z becomes a imaginary number and it's hard to define the independent components $(E_{s;m}, E_{p;m})$. Here, as we stated in the main-text, for an arbitrary diffraction order, we use the components $(E_{x,m}(\text{NF}), E_{y,m}(\text{NF}))$ to depict the near-field polarization, whose complex amplitudes are $(c^n_{x,m}, c^n_{y,m})$ and NF denotes the position of the near field. According to the transverse condition Eq. S6, $E_{z,m}(\text{NF})$ can be determined by components $(E_{x,m}(\text{NF}), E_{y,m}(\text{ZF})$. In other words, $(E_{x,m}(\text{NF}), E_{y,m}(\text{NF}))$ can represent the near-field polarization of mth diffraction order in whole Brillouin zone (BZ).

2 Formation of lattice charge during BZ folding around BZ center

In this section, we present a theory to depict the formation of nontrivial lattice charge due to BZ folding. We consider a 1D PC slab with y-mirror symmetry first, in which the E_x component is absent along the k_x axis for TE-polarized modes. According to the CWT framework, near the 2nd Γ point, the 1st and -1st orders of diffraction dominate the energy inside the slab, and thus we refer to them as the basic orders. Other diffraction orders can be "excited" by the basic orders, and the physics of wave coupling can be concluded as an eigenvalue problem:

$$\begin{bmatrix} \omega_0 + \xi k_x + \eta k_y^2 & \Delta_x + \Delta_y k_y^2 \\ \Delta_x + \Delta_y k_y^2 & \omega_0 - \xi k_x + \eta k_y^2 \end{bmatrix} \begin{bmatrix} A_1 \\ A_{-1} \end{bmatrix} = \lambda \begin{bmatrix} A_1 \\ A_{-1} \end{bmatrix} \tag{S7}$$

namely:

$$\mathcal{H} V = \lambda V \tag{S8}$$

in which matrix $\mathcal{H}$ describes the coupling between two basic orders whose amplitudes are A_1 and A_{-1}. By solving Eq.S8, we can obtain the basic orders and further calculate other diffraction orders accordingly. In particular, the 0th diffraction order is derived as:

$$V_0 = \mathcal{P}_0(k_x, k_y) V \tag{S9}$$

where $V_0 = [c^n_{x,0}, c^n_{y,0}]^T$ represents the amplitudes of 0th diffraction in the near-field, and $\mathcal{P}_0$ is the projecting matrix between the basic orders and 0th diffraction.

In CWT, the basic orders can be understood as the counter-propagating waveguide modes in a homogeneous slab with permittivity of ξ_0, governed by:

$$\left[\frac{\partial^2}{\partial z^2} + \xi_0 \omega_\pm(\mathbf{k}_{/\!/})^2 - \beta_\pm(\mathbf{k}_{/\!/})^2\right] \Theta_\pm(\mathbf{k}_{/\!/}, z) = 0 \tag{S10}$$

Around the second Γ point, due to the phase-matching condition, it's clear that here basic order of $m = -1$ is exactly the right-propagating waveguide mode (G^1_R) and basic order of $m = 1$ is the left-propagating one (G^1_L). Therefore, we have $\beta_\pm(\mathbf{k}_{/\!/})^2 = [(\pm 1 - k_x)^2 + k_y^2]\beta_0^2$, which are the in-plane wavenumbers of propagating modes with lower scripts + and − denoting the left- and right-propagating mode, respectively.

Eq. S7 indicates that the Bloch modes come from the diffraction of periodic lattice that couples the waveguide modes (basic orders) with other orders. Accordingly, the parameters in Eq. S7 can be interpreted as

follows: $\omega_0 = \omega_{\pm}(\Gamma)$ is the degenerate frequency of waveguide modes at Γ point, ξ and η describe their dispersion in momentum space, and $\Delta_{x,y}$ describe the coupling between the two waveguide modes where other diffracted orders can be treated as the coupling routes. When the PC slab degrades to a homogeneous slab, $\Delta_{x,y}$ vanish, and other diffraction orders disappear (i.e. $\mathcal{P}_0 = 0$) in spite of the basic ones.

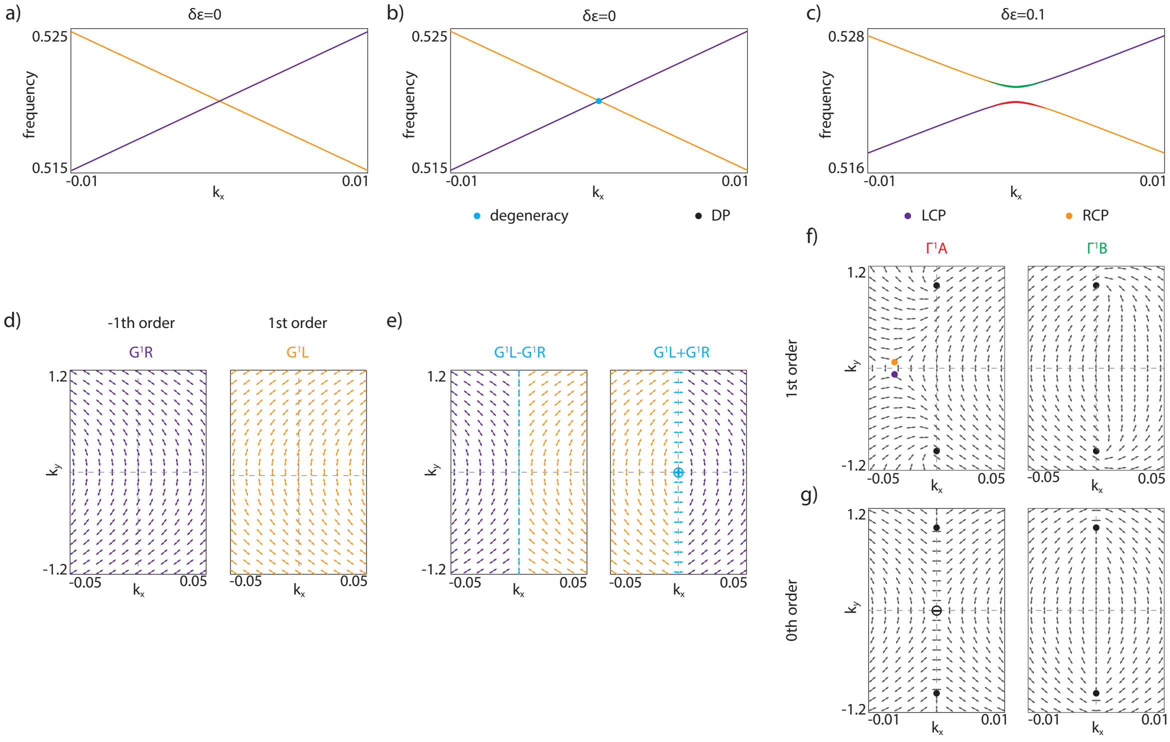

Figure S1: **Spontaneous symmetry breaking and formation of lattice charge.** (a,d) the band structures, polarization vector fields of the propagating waveguide modes G_L^1 and G_R^1 for $\delta\varepsilon = 0$. (b,e), the band structures, polarization vector fields of $G_L^1 \pm G_R^1$ under hybridization at $k_x = 0$. (c,f,g) the band structures, polarization vector fields of 1st and 0th diffraction orders of the band edge modes Γ_A^1 and Γ_B^1 for $\delta_\varepsilon = 0.1$.

First, we consider a homogeneous slab with $\delta_\varepsilon = 0$. Other parameters are the same as the Fig.3 in the main-text. In this case, according to Eq. S7, two eigenvectors can be solved as $[1 \;\; 0]^T$ for G_L^1 and $[0 \;\; 1]^T$ for G_R^1, which is valid for every $\mathbf{k}_{//}$ in momentum space. By solving Eq. S10, their band structures along k_x axis are shown in Fig. S1a. Clearly, the two waveguide modes cross at the Γ point. Note that this crossing is intrinsically caused by BZ folding. According to the definition of near-field polarization we present in the main-text, we calculate the polarization vector fields of these two waveguide modes accordingly:

$$c^n_{\pm,x} = \frac{k_y}{\sqrt{(\pm 1 - k_x)^2 + k_y^2}}\Theta_\pm(\mathbf{k}_{/\!/}, \mathrm{NF}) \qquad c^n_{\pm,y} = \frac{\pm 1 - k_x}{\sqrt{(\pm 1 - k_x)^2 + k_y^2}}\Theta_\pm(\mathbf{k}_{/\!/}, \mathrm{NF}) \tag{S11}$$

The resulted polarization fields are plotted in Fig. S1d, showing no polarization defects are found.

G^1_L and G^1_R are degenerate along the whole k_y axis (blue dot, Fig. S1b), forming a line degeneracy where they can be hybridized in any ratio. This line degeneracy is a direct consequence of the BZ folding, and corresponds to the singularity of eigenmode hybridization (mode singularity). Here, we choose the linear combinations of G^1_L and G^1_R: $G^1_L \pm G^1_R$, namely $[1 \;\; 1]^T$ and $[1 \;\; -1]^T$ at $k_x = 0$, which are actually the eigenvectors at the Γ point with $\delta_\varepsilon \neq 0$. In other words, the linear combination is consistent with the Bloch modes after the BZ folding and periodic permittivity modulation, known as the spontaneous symmetry breaking.

By applying the hybridization, we restructure the eigenvectors along the k_y axis, distinguish the bands by their frequencies, and replot the polarization field in Fig. S1e. Obviously, no charge is found in the $G^1_L - G^1_R$ band ($q^n_- = 0$), but a nonzero charge of $q^n_+ = +1$ emerges for $G^1_L + G^1_R$ band. This charge q^n_+ doesn't correspond to any realistic polarization defects in diffraction orders, because here we still consider a homogeneous slab. It indeed comes from the spontaneous symmetry breaking of the line degeneracy at $k_x = 0$, and represents the mode singularity due to BZ folding. Therefore we refers to it as the "lattice charge". Since the lattice charge is a representation of the mode singularity, it turns out to be real polarization defects at diffraction orders when realistic periodic modulation is introduced.

Further, we consider a notable periodic permittivity modulation $\delta_\varepsilon = 0.1$ to lift the line degeneracy (Fig. S1c), and two waveguide modes get anti-crossed to split to two band edge modes: Γ^1_A (highlighted in red) and Γ^1_B (highlighted in green). In this case, the diffraction is realistic, and the lattice charge creates nontrivial polarization defects on every diffraction orders. As examples, we plot the polarization fields of 1st and 0th diffraction orders, respectively (Fig. S1f and g). The same as Fig. 3c in the main-text, a pair of Dirac point (DPs) emerge, each carrying a half-charge of $q^n_1 = 1/2$ (black dots). Besides, two flying CPs emerge at ($k_x = -0.0198, k_y = \pm 0.002$) in the 1st diffraction of Γ^1_A band, each carrying half-charges of $q^n_1 = -1/2$. As a result, the total charges on the 1st diffraction for Γ^1_A and Γ^1_B are conserved with the lattice charges: $q^n_{1,A} = 1/2 + 1/2 - 1/2 - 1/2 = 0 = q^n_-$ and $q^n_{1,B} = 1/2 + 1/2 = 1 = q^n_+$. Similarly, the conservation can be found for the 0th diffraction too. Note that the integer defect at Γ point for 0th diffraction of Γ^1_A mode

represents a symmetry-protected BIC. Consequently, we calculate $q^n_{0,A} = 1/2 + 1/2 - 1 = 0 = q^n_-$ and $q^n_{0,B} = 1/2 + 1/2 = 1 = q^n_+$, which are the same with the lattice charge.

To sum up, we argue that the lattice charge comes from eigenvector hybridization at the line degeneracy caused by BZ folding, and are determined from both the waveguide modes as well as the lattice geometry – the latter determines how the BZ folds. When realistic permittivity modulation lifts the degeneracy, it turns to be real polarization defects in every diffraction order. The specific unit-cell geometry determines how lattice charges turn into realistic polarization defects. As an example shown in Fig. S1f and g, in spite of the half defects carried by the DPs, the lattice charge on the 1st diffraction creates a pair of half-charges carried by CPs, while the lattice charge on 0th diffraction creates a symmetry-protected BIC protected by the C_2 symmetry. In other words, the symmetry-protected BIC is a consequence of lattice charge combined with the C_2 symmetry, which is only available at the Γ point. If we break the C_2 symmetry of the unit cell, the integer charge on 0th diffraction would split to a pair of half-charges carried by the CPs, too, the same as we observe in the 1st diffraction.

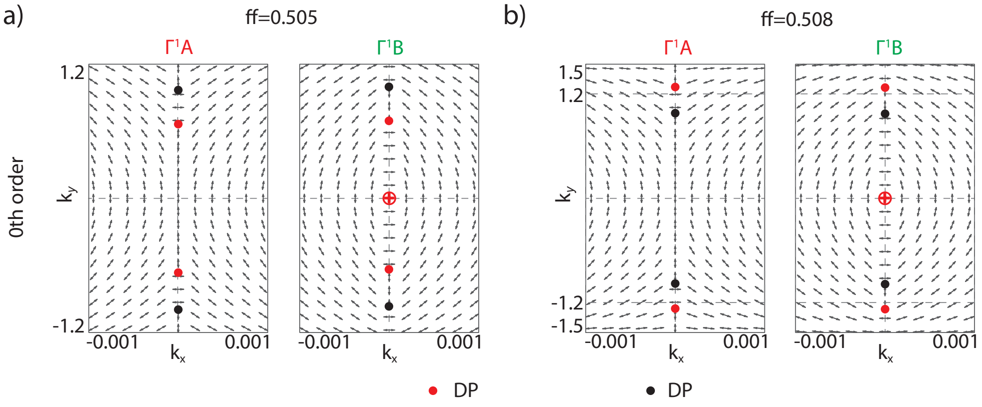

Figure S2: **Lattice charge conservation.** a,b, 0th charges configuration for both Γ^1_A and Γ^1_B bands when ff = 0.505 and ff = 0.508, respectively.

Another example is shown in Fig. S2, where we calculate the charge configurations with different filling factors (ff = r/a). Comparing to Fig. S1g with ff = 0.4, when ff = 0.505 (Fig. S2a), the two Bloch modes at Γ point reveres, and thus the BIC turns to appear at Γ^1_B band, carrying 0th integer charge of $q^n_0 = +1$. At the same time, another pair of DPs emerge along k_y axis (red dots), each carrying a 0th half charge of $q^n_0 = -1/2$. As a result, the total 0th charge for Γ^1_A is $q^n_{0,A} = 1/2+1/2-1/2-1/2 = 0$ and the the total charge for Γ^1_B is $q^n_{0,B} = +1 + 1/2 + 1/2 - 1/2 - 1/2 = 1$, both conserved with the lattice charge shown in Fig. S1.

Further, if we continue increasing the filling factor, the new-born DP pair continues departing away from Γ point, as shown in Fig. S2b. However, the total 0th charges are the same. This example indicates that, when unit-cell geometry changes, the specific topological charge configuration may change, but the total charges including varieties of generated polarization defects are conserved to the lattice charge.

3 Formation of lattice charge during BZ folding around BZ edge

As we stated in the main-text, the BZ folding can also happen at BZ edge, giving rise to the formation of lattice charge, too. Specifically, as shown in Fig. 1b in the main-text, we consider the folding of waveguide mode G^1 at the BZ edge, which gives rise to two band-edge bloch modes Γ^1_A and X^1_B (blue circle, Fig. 1).

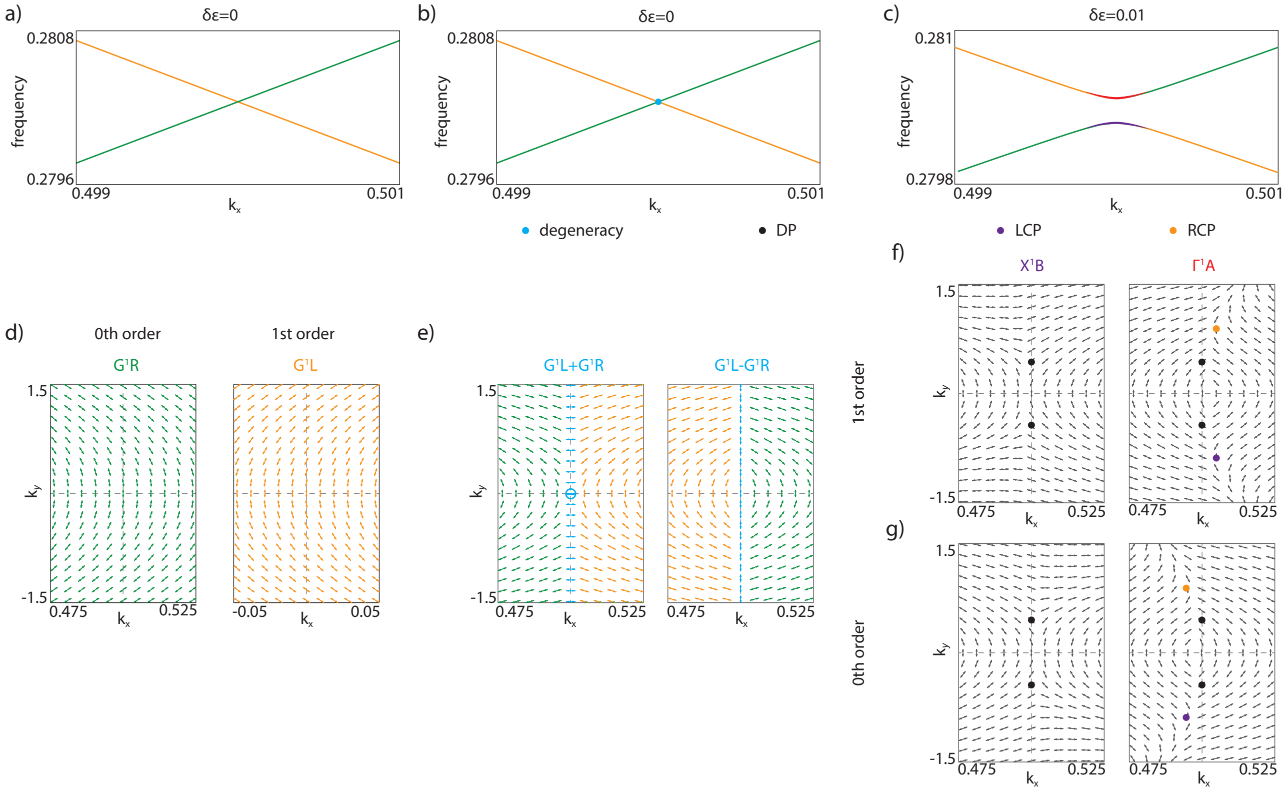

Figure S3: **Formation of lattice charge at BZ edge.** (a,d) the band structures, polarization vector fields of the propagating waveguide modes G^1_L and G^1_R for $\delta_\varepsilon = 0$. (b,e), the band structures, polarization vector fields of $G^1_L \pm G^1_R$ under hybridization at the Γ point. (c,f,g) the band structures, polarization vector fields of 1st and 0th diffraction orders of the band edge modes Γ^1_A and X^1_B for $\delta_\varepsilon = 0.01$.

We follow the same procedures as we did in the last section. We apply the CWT framework around the first X point, and choose the basic orders as 0th ($m = 0$) and 1st ($m = 1$) diffraction, correspond to the waveguide modes G^1_R and G^1_L, respectively. When periodic permittivity modulation is zero ($\delta\varepsilon = 0$), the band structures and polarization vector fields of G^1_{RL} are calculated according to Eq.S10 and Eq.S11, as shown in Fig. S3a and d, respectively. No polarization defects can be found.

Similar with the case around Γ point, G_R^1 and G_L^1 are degenerate at $k_x = 0.5$, forming a line degeneracy resulted from the BZ folding. Spontaneous symmetry breaking of such a line degeneracy creates lattice charge of $q_+^n = -1$ for $G_L^1 + G_R^1$ and $q_-^n = 0$ for $G_L^1 - G_R^1$ (Fig. S3b and e). By introducing realistic permittivity modulation ($\delta\varepsilon = 0.01$), the line degeneracy lifts to form two band-edge modes around X point: Γ_A^1 and X_B^1 (Fig. S3c), and the lattice charges turn to be realistic polarization defects carrying nonzero topological charges on every diffraction orders. The generated charge configurations of 0th and 1st diffraction orders for both two bands are shown in Fig. S3f and g. The same, the total charge is conserved to the lattice charge.

There are several interesting facts. First, 0th diffraction order and 1st diffraction are symmetric to each other with respect to the $k_x = 0.5$ axis, so their polarization fields and polarization defects distribution are also symmetric to each other. Second, at BZ edge, none of diffraction orders are protected by the C2 symmetry. Therefore, half charges carried by flying CPs are generated rather than the integer charge. Third, around both Γ point and X point, the lattice charge for linear combination $G_L^1 - G_R^1$ is always $q_-^n = 0$, both corresponding to the Γ_A^1 mode. This again indicates that the lattice charge is independent with the specific symmetry. In fact, around Γ point and X point, the symmetry of Γ_A^1 mode are different so the generated polarization defects are also different. But, the lattice charge is unchanged. On the other hand, the lattice charge for $G_L^1 + G_R^1$ is different around Γ point and X point. Around Γ point, $G_L^1 + G_R^1$ corresponds to the Γ_B^1 mode, while around X point it corresponds to the X_B^1 mode.

4 Lattice charge in 2D photonic crystal slab

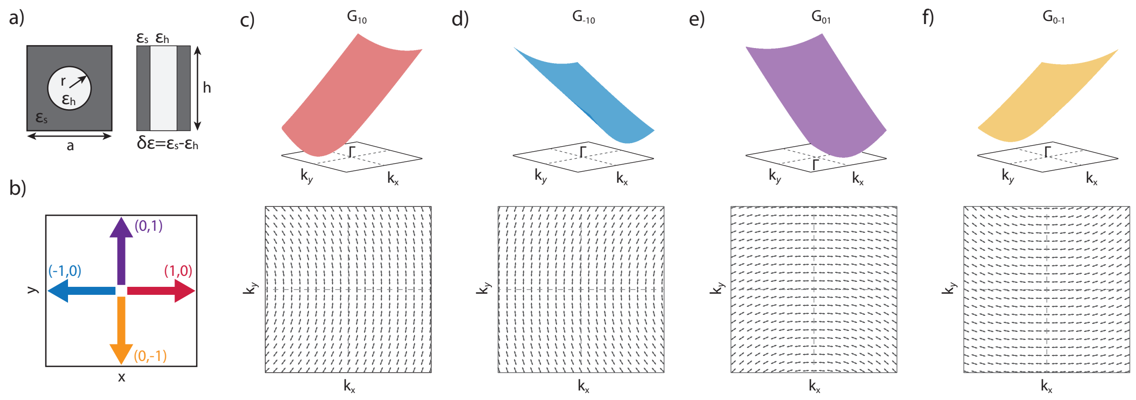

Figure S4: **Waveguide modes in 2D PC slab** (a) schematic of 2D PC slab. $a = 500$ nm; $r = 150$ nm; $h = 220$ nm; $\varepsilon_s = 4.0804$. (b) schematic of four propagating waveguide modes in a 2D homogeneous slab. (c,d,e,f) the band structures (upper panels) and polarization vector fields (lower panels) of the propagating waveguide modes P_{10}, P_{-10}, P_{01}, P_{0-1}, respectively.

According to Eq.S1, for TE-polarized Bloch modes in a 2D PC slab (Fig. S4a), we use the index pair (m, n) to denote the diffraction orders. In the vicinity of 2nd Γ point, four diffraction orders dominates the energy: $(1, 0)$, $(-1, 0)$, $(0, 1)$ and $(0, -1)$, and we refer them as the basic orders. The basic orders correspond to four propagating waveguide modes in the 2D homogeneous slab, denoted as G_{10}, G_{-10}, G_{01} and G_{0-1} (Fig. S4b). By solving the wave equations similar to Eq. S10, we calculate the band structures and polarization vector fields of the four waveguide modes, shown in Fig. S4c,d,e, and f, respectively. Obviously, for each waveguide mode, no polarization defects can be found.

The propagating waveguide waves would be diffracted by the periodic lattice into other diffraction orders, and thus, they couple with each other to form four Bloch bands, leading to an eigenvalue problem:

$$\begin{bmatrix} \omega_0 + \xi k_x + \eta k_y^2 & b & c & c \\ b & \omega_0 - \xi k_x + \eta k_y^2 & c & c \\ c & c & \omega_0 + \eta k_x^2 + \xi k_y & b \\ c & c & b & \omega_0 + \eta k_x^2 - \xi k_y \end{bmatrix} \begin{bmatrix} A_{10} \\ A_{-10} \\ A_{01} \\ A_{0-1} \end{bmatrix} = \lambda \begin{bmatrix} A_{10} \\ A_{-10} \\ A_{01} \\ A_{0-1} \end{bmatrix} \tag{S12}$$

where ω_0 is the degenerate frequency of four waveguide modes at the Γ point; ξ and η are dispersion coefficients of freely propagating waveguide modes, whose amplitudes are denoted as A_{10}, A_{-10}, A_{01} and A_{0-1}; b and c describe the coupling strength between the waveguide modes (basic orders) and other diffraction

orders. For a homogeneous slab with $\delta_\varepsilon = 0$, we have $b = c = 0$, and the four waveguide modes can be represented by the eigenvectors:

$$\begin{aligned} G_{10} &= [1\ 0\ 0\ 0]^T \qquad G_{-10} = [0\ 1\ 0\ 0]^T \\ G_{01} &= [0\ 0\ 1\ 0]^T \qquad G_{01} = [0\ 0\ 0\ 1]^T \end{aligned} \tag{S13}$$

While for a lattice with nonzero δ_ε, four band-edge modes can be solved at the Γ point as:

$$\begin{aligned} TE_A &= [1\ 1\ 1\ 1]^T \qquad TE_B = [1\ 1\ -1\ -1]^T \\ TE_C &= [1\ -1\ 0\ 0]^T \qquad TE_D = [0\ 0\ 1\ -1]^T \end{aligned} \tag{S14}$$

To show how the lattice charges are generated in a 2D lattice, we also start from a homogeneous slab ($\delta_\varepsilon = 0$) and its band structures are illustrated in Fig. S5a, showing that the waveguide modes intersect with each other. We distinguish the four bands from their frequencies (upper panels, Fig. S5c-f), showing that each band is composed of the four waveguide modes and includes several intersection lines (green lines). Along the intersection lines, the waveguide modes are degenerate and can be hybridized with each other. Specifically, we take the TE_A band as an example. According to Eq. S14, the superposition coefficients can be chosen as $[1111]^T$, and thus the phase diagram (upper panel, Fig. S5c) indicates that every waveguide mode has the same weight (denoted by marker +). On each intersection line, the mode should be a hybridization of the two weighted waveguide modes in the adjacent regions. For example, on the intersection line between the red region (G_{10}) and purple region (G_{01}), the linear combination is $[1010]^T$. And then, We restructure the band and plot the polarization vector fields in middle panel of Fig. S5c, showing that a lattice charge of $q_0^n = +1$ can be found.

A similar process happens for the other three bands, and we can calculate the lattice charge correspondingly: $q_0^n = -1$ for TE_B band; $q_0^n = +1$ for the $TE_{C,D}$ bands. After applying a notable permittivity modulation, such lattice charges would turn into realistic polarization defects in diffraction orders. For example, we consider a 2D lattice with $\delta_\varepsilon = 0.5$ to create four band-edge modes residing near the 2nd Γ point (Fig. S5b), where TE_A and TE_B are isolated bands while TE_C and TE_D are degenerate at the Γ point. Protected by C_4 symmetry, the lattice charges on TE_A and TE_B exhibit as symmetry-protected BICs that carry integer defects in the 0th diffraction. As for the degenerate modes TE_C and TE_D, the lattice charge shows as a polarization defect due to the degeneracy (green dot) with a topological charge of $q_0^n = -1$. Besides, four pairs of EPs are found on

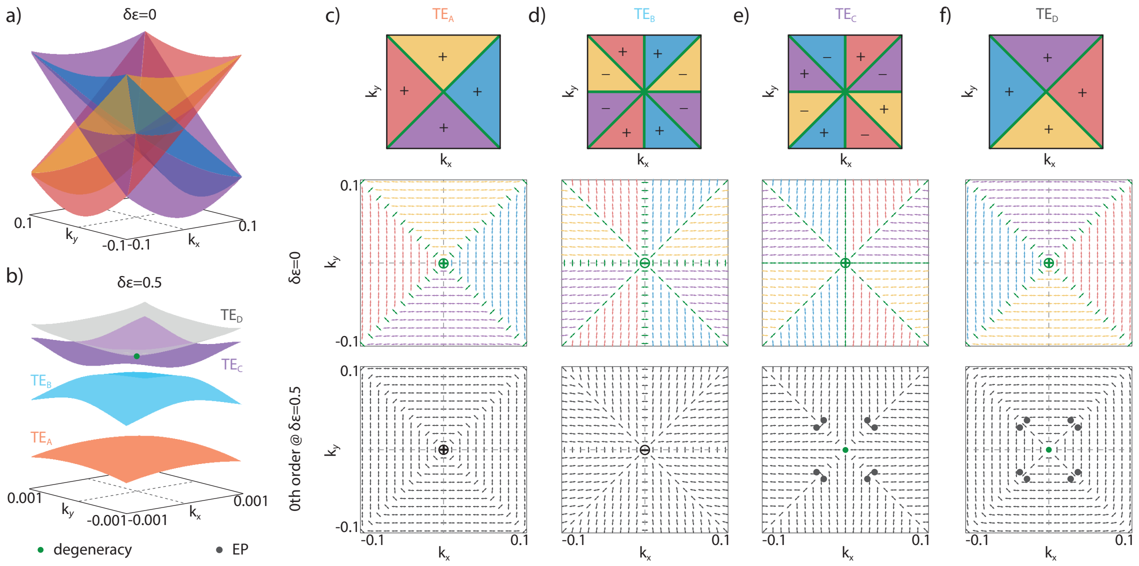

Figure S5: **Lattice charge generation in a 2D PC slab.** (a) band structures of four propagating waveguide modes of $\delta_\varepsilon = 0$. (b) band structures of four band edge modes of $\delta_\varepsilon = 0.5$. (c,d,e,f) phase diagram (upper panel), polarization vector field under hybridization at $\delta_\varepsilon = 0$ (middle panel), and polarization vector field of 0th diffraction for four band-edge modes$\delta_\varepsilon = 0.5$ (lower panel).

TE$_C$ and TE$_D$ bands along the M direction, each carrying a charge of $q_0^n = 1/2$. Summing them up, the total charges on TE$_C$ and TE$_D$ bands are $q_{0,C}^n = q_{0,D}^n = -1 + 1/2 \times 4 = +1$, which is conserved to the original lattice charge.

As we emphasized in the last section, the unit-cell geometry determines how lattice charges turn into realistic polarization defects. In 2D PCs, the behaviors are more complicated. As for the specific PC slab shown in Fig. S4a, first, we can change the modes order at Γ point by tuning the parameters such as filling factor or permittivity: the bands may flip and thus the TE$_B$ and TE$_C$ modes can be degenerate while the TE$_A$ and TE$_D$ become isolated. In this way, the lattice charge on the TE$_D$ band may become a BIC. Also, we can break the C$_4$ symmetry into C$_2$ to lift the degeneracy between TE$_C$ and TE$_D$ modes. In this way, the degenerate point at Γ point would crash into a pair of EPs. If we further break the C$_2$ symmetry, the symmetry-protected BICs on TE$_A$ and TE$_B$ bands would also split into CP pairs. In a word, complicated defects configuration can be created by choosing different unit-cell geometries, but the total charges carried by varieties of polarization defects are conserved to the original lattice charge.

5 Details of polarization defects generated from accidental band crossings around the X point

As a supplement to Fig. 4 in the main text, we discuss the details of polarization defects generated from accidental band coupling between Γ_A^1 and X_B^3 at the X point. The band crossing scenario of X_B^3 and Γ_A^1 at the X point is shown in Fig. S6a. As we stated in the main-text, right at the DP, the amplitudes of diffraction orders are ill-defined due to modes degeneracy, which can be verified from Fig. S6b. The trajectories of the 0th polarization defects carrying integer topological charge (Fig. 4b in the main text) are directly derived from Fig. S6b, showing that they are spawned from the DP.

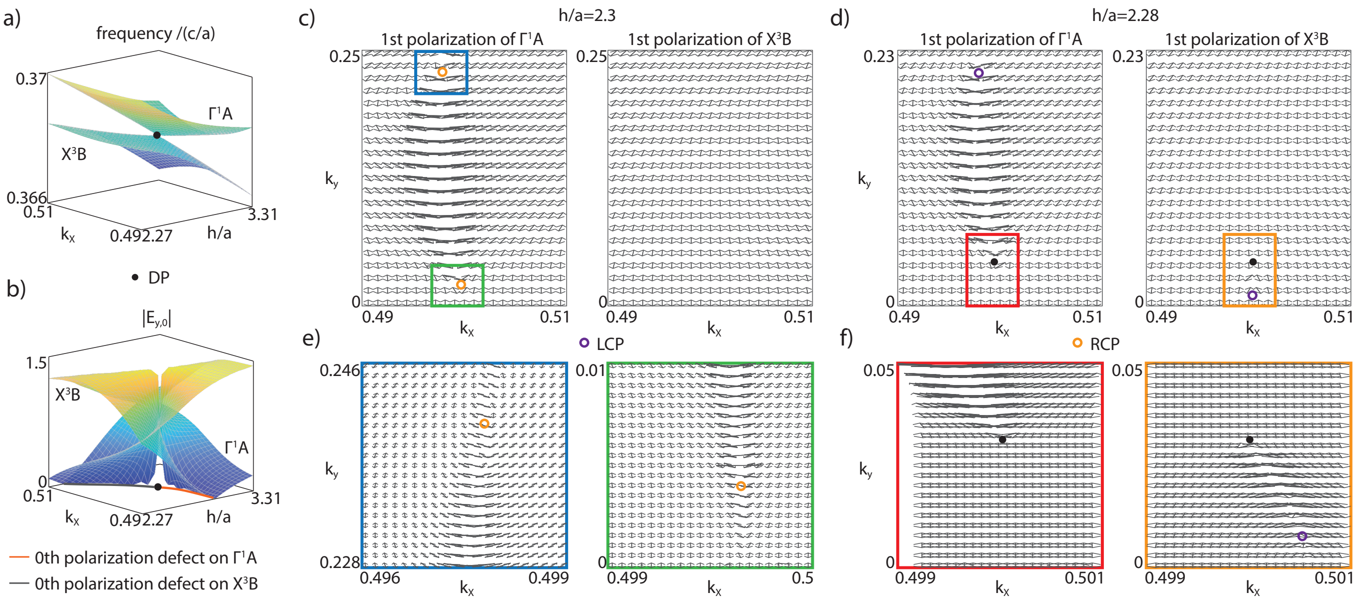

Figure S6: **Supplementary figure to Fig. 4 in the main-text.** (a,b) band structures and 0th diffraction amplitude of the Γ_A^1 and X_B^3 modes near the X point in parameter space (k_x, h) when $k_y = 0$. black dot: DP; red/gray line: trajectory of 0th polarization defect in Γ_A^1/ X_B^3 mode. (c,d) polarization vector fields of 1st diffraction order of Γ_A^1 and X_B^3 modes when $h/a = 2.3$ and $h/a = 2.28$. (e) zoom-in regions in blue and green boxes in (c). (f) zoom-in regions in red and orange boxes in (d).

Not only on the 0th diffraction, the DP also creates defects on other diffraction orders. As shown in Fig. S6c, for $h/a = 2.3$ that no coupling happens between Γ_A^1 and X_B^3 modes (Fig. 4c in the main-text), a pair of CPs carrying opposite half-charges (orange circles, Fig. S6e) emerges on the 1st diffraction of Γ_A^1 band in the region of interest (ROI) while no charge are found in X_B^3 band. Note that, the total topological charges in the ROI are counted as zero for both Γ_A^1 and X_B^3 bands on the 1st diffraction.

By further decreasing the slab thickness to $h/a = 2.28$, Γ_A^1 and X_B^3 bands get coupled and give rise to two DPs at ($k_x = 0.5, k_y = \pm 0.03$), which is the same with DPs in 0th diffraction (Fig. 4f in the main text). In this case, two DPs correspond to non-zero half-charges of $q_1^n = -1/2$ on the 1st diffraction, as shown in Fig. S6d and f. Despite the DPs, there also exist two CPs carrying half-charges of $q_1^n = 1/2$ (purple circles) on both Γ_A^1 and X_B^3 bands, respectively. As a result, the total topological charges on the 1st diffraction remain as conserved numbers in the ROI during the inter-band coupling: namely $q_{A,1}^n = q_{B,1}^n = 1/2 - 1/2 = 0$. Similar to the topological charges on 0th diffraction, the conservation law here is also a direct consequence of Stokes' theorem.

We emphasize that, beyond the ROI, there may exist other CP pairs carrying non-zero charges because the reduced BZ is not a compact manifold for any diffraction orders. However, once we choose an ROI, according to Stokes' theorem, the local conservation of total topological charges inside the ROI always holds unless nonzero topological charges cross the boundary of the ROI.

6 Details of polarization defects generated from accidental band crossings around the Γ point

In this section, we present a complete evolution of all types of polarization defects during the interband coupling between Γ_A^3 and Γ_B^1 when they accidentally cross with each other, shown in the dashed blue circle in Fig. 1b in the main-text. The coupling scenario between Γ_A^3 and Γ_B^1 bands is shown in Fig. S7a and b, in which paired EPs can be observed as the branching points of the complex Riemann sheets. The paired EPs and the secant line connecting them are shown in Fig. S7c, which divides the parameter space into several regions (dashed lines). We focus on the 0th diffraction in this section and discuss the polarization defect evolution across the mentioned regions.

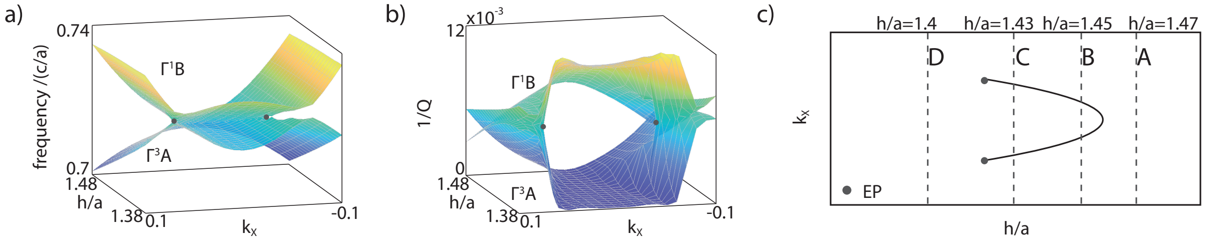

Figure S7: **Coupling scenario of Γ_A^3 and Γ_B^1 bands** (a,b) interband coupling between Γ_A^3 and Γ_B^1 in the 2D parameter space (k_x, h). (c) Schematic of the EP pair and Fermi arc in parameter space.

For $h/a = 1.47$ that no coupling happens between Γ_A^3 and Γ_B^1 bands (left panel, Fig. S8a), only an integer defect of $q_0^n = 1$ can be found in the Γ_A^3 band corresponding to a symmetry-protected BIC (right panel, Fig. S8a), but there doesn't exist any polarization defect in the Γ_B^1 band (middle panel, Fig. S8a). When slab thickness decreases to $h/a = 1.45$, Γ_A^3 and Γ_B^1 bands simply cross without coupling (left panel, Fig. S8b). In this case, we still cannot find any polarization defects in the Γ_B^1 band (middle panel, Fig. S8b). However, in the Γ_A^3 band, the integer defect representing the symmetry-protected BIC turns to be $q_0^n = -1$ rather than its origin value of $q_0^n = 1$ in Fig. S8a, accompanied by four flying CPs each carrying a negative half-charge (right panel, Fig. S8b). Nevertheless, we check the total topological charges for the Γ_A^3 band and find it is still a conserved quantity as $q_{A,0}^n = -1 + 1/2 \times 4 = +1$, which is the same as the case of $h/a = 1.47$.

We further decrease the slab thickness to $h/a = 1.43$ to make the Γ_A^3 and Γ_B^1 bands coupled to each other and generate four pairs of EPs as shown in Fig. S8c. Because the two bands are flipped during band crossing, no integer defects (namely BICs) can be found in Γ_A^3 band (right panel, Fig. S8d). Instead, we find that two

EP pairs along the k_y axis (highlighted in blue) carry positive half-charges of $q_0^n = 1/2$, while the other two EP pairs along k_x axis (highlighted in gray) are trivial in the means of polarization defect. Again, we check the total topological charges are still conserved for the Γ_A^3 band as $q_{A,0}^n = 1/2 \times 2 = +1$. A similar argument can be applied to the Γ_B^1 band. As shown in the left panel of Fig. S8d, the symmetry-protected BIC carries an integer charge of $q_0^n = +1$, while the EP pairs in the k_y axis carry positive half-charges of $q_0^n = +1/2$. Besides, there exist 8 flying CPs and each carries negative half-charges of $q_0^n = -1/2$, and 4 flying CPs carry positive half-charges of $q_0^n = +1/2$. Therefore, the total topological charge of the Γ_B^1 band are counted as $q_{B,0}^n = +1 + 1/2 \times 2 - 1/2 \times 8 + 1/2 \times 4 = 0$, which is conserved to the uncoupled case.

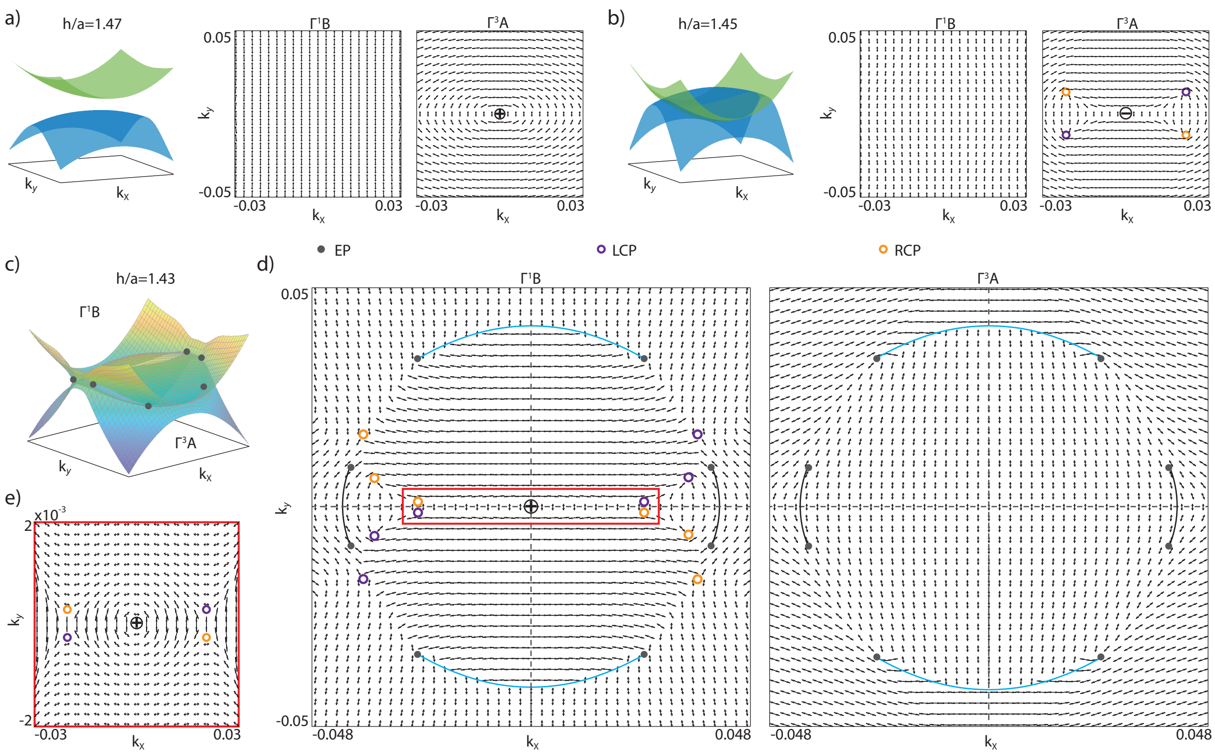

Figure S8: **Detailed configuration of polarization defects emerging during interband coupling between Γ_A^3 and Γ_B^1 modes.** (a,b) band structure, polarization vector fields of Γ_A^3 and Γ_B^1 modes in momentum space when $h/a = 1.47$ and $h/a = 1.45$, respectively. (c) band structure when $h/a = 1.43$. (d) polarization vector fields of Γ_A^3 and Γ_B^1 modes in momentum space when $h/a = 1.43$. (e) zoom-in region highlighted in red.

To summarize, we notice many polarization defects may emerge during the interband coupling process, including flying CPs with non-zero half-charges, EPs that may or may not carry non-zero half-charges, and the

symmetry-protected BIC with integer charge which can change its sign. Nevertheless, the total topological charge on both Γ_A^3 and Γ_B^1 bands are always conserved, no matter how complicated the topological charge configuration is.

In fact, a FW-BIC can be generated from the interband coupling between Γ_B^1 and Γ_A^3, too. As shown in Fig. S9, when we continue decreasing the slab thickness to about $h/a = 1.391$, two pairs of CPs are spawn from the void. Then, two CPs carrying the same charge of $q_0^n = 1/2$ move towards each other and finally emerge to form an integer charge (Fig. S9d), giving rise to the so-called FW BIC. During this process, it doesn't violate such conservation if we take the two accompanying flying CP into account. Note that in Fig. Fig. S9e, we neglect two CPs and zoom-in the region around the integer charge. The total charge upon the ROI is conserved to zero during the whole process.

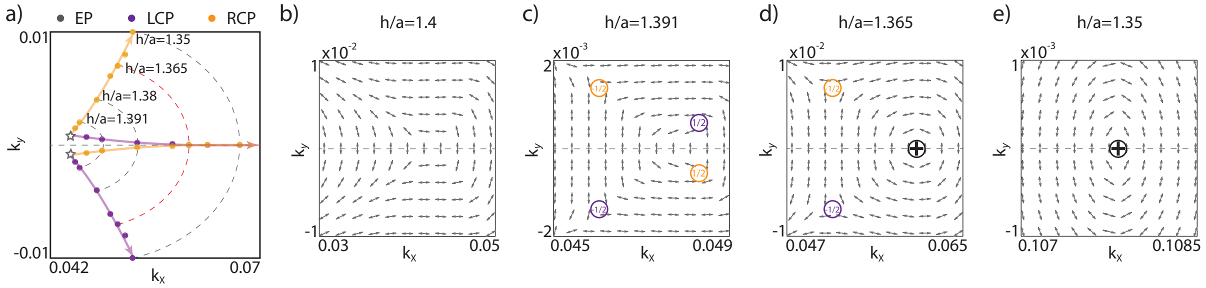

Figure S9: **Generation of FW BIC from the interband coupling between Γ_A^3 and Γ_B^1 modes.** (a) Evolution trajectories of CPs spawn from the void during the interband coupling between Γ_B^1 and Γ_A^3 when varying the slab thickness. (b,c,d,e) topological charge configuration in momentum space when $h/a = 1.4,\ 1.391,\ 1.365,\ 1.35$.

Another interesting finding is that the EP pair doesn't necessarily carry non-trivial topological charges. Actually, the half-charges of an EP pair are intrinsically generated from the flipped two bands with orthogonal polarization. Notice the fact that a loop path around the EP pair would evolve to another Riemann sheet then return back to the original Riemann sheet [2,3]. If the two sheets have orthogonal polarization, for example, Γ_A^3 and Γ_B^1 modes in the k_y axis, the polarization major axis would gain an extra phase of π by circling around the EP pair, and hence, exhibits as a half-charge. As a comparison, for an EP pair along the k_x axis, the two sheets are both y-polarized, and thus no extra phase would be obtained by circling such an EP pair, therefore no half-charge is generated. In other words, the possible half-charge carried by the EP pair doesn't result from its intrinsic property of band topology.

7 Polarization defects generated from the accidental band crossings in 2D PC slab

In this section, we discuss the alternative origin of polarization defects in 2D PC slab from the interband coupling between two crossed Bloch bands. Without loss of generality, we consider a PC slab (Fig. S10a) with Γ_A^1 and X_B^3 bands (Fig. S10b). The mode profiles at the X point are shown in Fig. S10c.

An integer charge on Γ_A^1 band can be found, corresponding to an accidental BIC. By tuning the slab thickness h/a from 1.1 to 2.8, the tunable BIC evolves along the k_x axis and smoothly crosses the light line. The trajectory (Fig. S10d) proves that the definition of near-field topological charge we propose in the main-text is also valid in the 2D case. Γ_A^1 and X_B^3 band get coupled and show an anti-crossing feature (Fig. S10b), and the full coupling scenario near the X point is presented in Fig. S10e. Similar to the 1D case in Fig. 4a in the main-text, a DP can be found at ($k_x = 0.5, k_y = 0, h = 0.2785a$), marked by a black dot. Integer charges on both Γ_A^1 and X_B^3 bands are spawned from the DP, verified by their trajectories shown in Fig. S10f. This example indicates that the intrinsic physics of generating polarization defects from the accidental band crossings is quite the same as the 1D case.

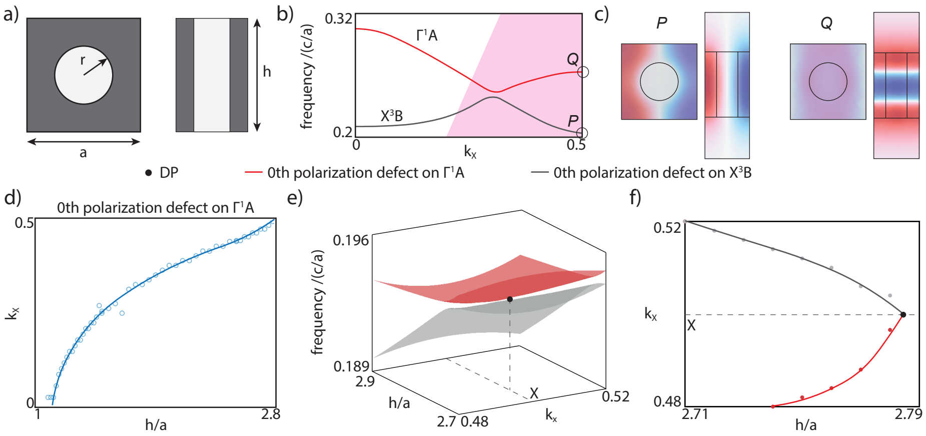

Figure S10: **Polarization defect from accidental band crossing in a 2D PC slab** (a) schematic of the 2D PC slab. a = 720 nm; r = 180 nm; slab permittivity is 12.96. (b) band structures of Γ_A^1 and X_B^3 modes. (c) model profiles of Γ_A^1 and X_B^3 at the X point. (d) trajectory of a 0th integer charge on Γ_A^1 band along k_x axis when h varies. (e) coupling scenario between Γ_A^1 and X_B^3 bands around the X point. (f) trajectories of integer charges on both Γ_A^1 and X_B^3 bands in 2D parameter space (k_x, h) when $k_y = 0$.

8 Polarization defects in unfolded 2D BZ

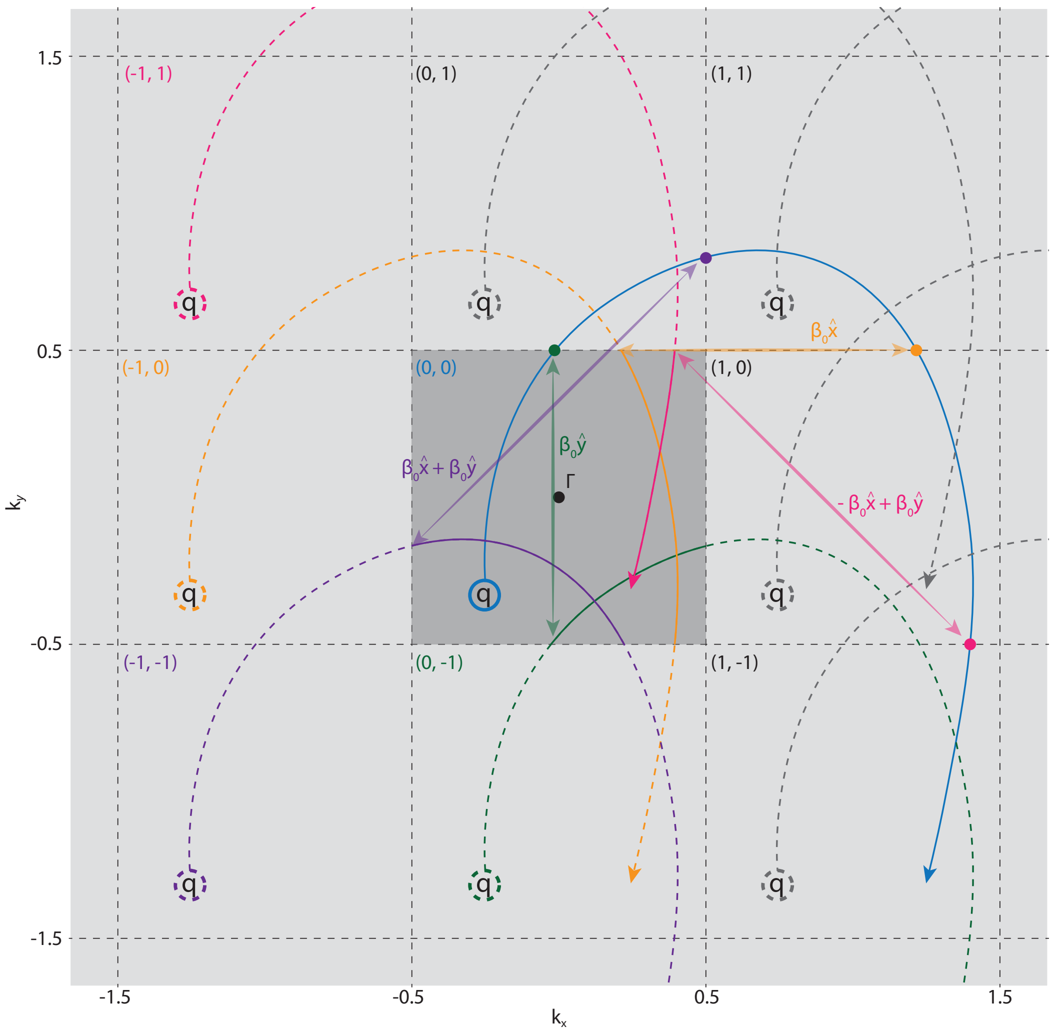

Figure S11: **Polarization defects in unfolded 2D BZ.** Dark grey shading: reduced BZ; Light grey shading: entire BZ; Solid arrows: evolution trajectories of polarization defects in different diffraction orders.

In this section, we present the configuration of polarization defects in unfolded 2D BZ. As shwon in Fig. S11, in 2D PC slab, the order of diffraction is labelled by two indices: (m, n), with the in-plane wavevector of $\mathbf{g_{m,n}} = (m - k_x)\beta_0\hat{e_x} + (n - k_y)\beta_0\hat{e_y}$. Similar with the 1D case, increasing or decreasing the order of diffraction (m, n) by (Δ_m, Δ_n) is equivalent to translating in the momentum space by reciprocal lattice vector of $(\Delta_m\beta_0\vec{e_x}, \Delta_n\beta_0\vec{e_y})$. Therefore, as we state in the main-text, there are two ways to depict the polarization defects in diffraction orders for periodic structure: polarization defects in multi diffraction orders but limited inside the reduced BZ, and polarization defects in only one order but spread over the entire BZ. In other

words, we can divided the whole momentum space into several regions labelling by indices (w, v). Each region is a duplicate of the reduced BZ. Every polarization defect of diffraction order (m, n) in the reduced BZ has a duplicate in region (w, v) with the order changed to $(m + w, n + v)$. For example, for polarization defect of order $(m = -1, n = -1)$ (solid purple line) in the reduced region, it has a duplicate in region $(w = 1, v = 1)$ with order $(m + w = 0, n + v = 0)$. Similarly, we can convert any polarization defect of diffraction order (m, n) in reduced BZ to the 0th defect, simply by translating it to region $(w = -m, v = -n)$.

In this way, for 2D BZ, we have the similar conclusions as the 1D case. The behaviour of the polarization defects in multi diffraction orders is equivalent to the behaviour of polarization defect in one order (i.e. 0th order) but spread in entire BZ. For such unbounded momentum space, we can create inifinite polarization defects by folding the BZ simply. Therefore, the total number of charges is countless

1. Liang, Y., Peng, C., Sakai, K., Iwahashi, S. & Noda, S. Three-dimensional coupled-wave model for square-lattice photonic crystal lasers with transverse electric polarization: A general approach. *Phys. Rev. B* **84**, 195119 (2011).

2. Zhen, B. *et al.* Spawning rings of exceptional points out of dirac cones. *Nature* **525**, 354–358 (2015).

3. Doppler, J. *et al.* Dynamically encircling an exceptional point for asymmetric mode switching. *Nature* **537**, 76–79 (2016).
